# A low-cost aeroponic phenotyping system for storage root development: unravelling the below-ground secrets of cassava (*Manihot esculenta*)

**DOI:** 10.1186/s13007-019-0517-6

**Published:** 2019-11-09

**Authors:** Michael Gomez Selvaraj, Maria Elker Montoya-P, John Atanbori, Andrew P. French, Tony Pridmore

**Affiliations:** 1 International Center for Tropical Agriculture (CIAT), A.A. 6713, Cali, Colombia; 2 School of Computer Science, University of Nottingham, Jubilee Campus, Wollaton Road, Nottingham, NG8 1BB UK; 3 School of Biosciences, University of Nottingham, Sutton Bonington Campus, Leicestershire, LE12 5RD UK

**Keywords:** Aeroponics, Auxin, Cassava, Dripponics, Semi-aeroponic, Root bulking, Storage root, Tuber crops

## Abstract

**Background:**

Root and tuber crops are becoming more important for their high source of carbohydrates, next to cereals. Despite their commercial impact, there are significant knowledge gaps about the environmental and inherent regulation of storage root (SR) differentiation, due in part to the innate problems of studying storage roots and the lack of a suitable model system for monitoring storage root growth. The research presented here aimed to develop a reliable, low-cost effective system that enables the study of the factors influencing cassava storage root initiation and development.

**Results:**

We explored simple, low-cost systems for the study of storage root biology. An aeroponics system described here is ideal for real-time monitoring of storage root development (SRD), and this was further validated using hormone studies. Our aeroponics-based auxin studies revealed that storage root initiation and development are adaptive responses, which are significantly enhanced by the exogenous auxin supply. Field and histological experiments were also conducted to confirm the auxin effect found in the aeroponics system. We also developed a simple digital imaging platform to quantify storage root growth and development traits. Correlation analysis confirmed that image-based estimation can be a surrogate for manual root phenotyping for several key traits.

**Conclusions:**

The aeroponic system developed from this study is an effective tool for examining the root architecture of cassava during early SRD. The aeroponic system also provided novel insights into storage root formation by activating the auxin-dependent proliferation of secondary xylem parenchyma cells to induce the initial root thickening and bulking. The developed system can be of direct benefit to molecular biologists, breeders, and physiologists, allowing them to screen germplasm for root traits that correlate with improved economic traits.

## Background

Cassava (*Manihot esculenta* Crantz), also referred to as manioc (French), yuca (Spanish), and other names in local regions, is a tropical root crop native to South America [1] which serves as a staple food source for more than 800 million people [2]. In tropical countries, cassava follows only maize and rice in its provision of calorie intake; about ten percent of the world’s population relies on it as their primary food source [3]. Its versatile nature—it is frequently referred to as the “drought, war and famine crop of the developing world” [4]—places it among the most adaptive crops during climate change. Despite its ability to withstand challenging environments, the slow progress of storage root (SR) bulking creates a long growth cycle (8–24 months), which can limit crop system options for farmers. Late storage root bulking is the single most crucial reason for rejection of cassava cultivars in most African countries [5]. Early storage root bulking in cassava is gaining more importance, particularly with smallholder farmers, whose economic situation is more fragile. Early-bulking varieties are urgently needed, which can be harvested in less than 8 months and at higher net returns compared to late-bulking types [6]. In recent times, early bulking is considered to have high potential to change cassava from the traditional ‘household food crop’ to an ‘industrial crop’ and therefore has become an important objective for the national cassava breeding programs in Africa and worldwide [7].

Overall plant productivity depends not only on storage roots but also on fibrous roots (FR), which are responsible for nutrient and water uptake and plant anchoring [8]. The growing insights into the genetic control of FR growth [9] can help augment cassava’s ability to explore and extract nutrients and water in limited resources environments [10]. There is an increasing number of published studies on the genetic, molecular and physiological regulation of root architecture as related to plant nutrient efficiency [11].

Root system architecture (RSA) and below-ground development have also received an increased amount of attention due to advances in root phenomics technologies [12, 13]. Although the importance of RSA in cereal crop productivity is well established, knowledge in root and tuber crops is still limited [14]. While RSA traits have been incorporated in root and tuber crop breeding programs for storage roots at mature plant stages (i.e. at harvest), traits for fibrous roots, or for storage roots during early development, have not. At present, storage roots are only assessed through destructive sampling which is very slow and labor-intensive. Because of multiple sources of variation, field-based or other destructive methods need to sample many replications of each genotype. This variation is due to the inherent difficulties of studying plant roots and the lack of a suitable model system for studying secondary growth in plant roots [15].

Although several factors, including environmental conditions and plant age, are known to influence plant trait variation in cassava, the role of genetic modulation of storage root initiation and bulking has not been explicitly investigated [16], since no protocol has been developed to visualize the storage root system over time under controlled-environment conditions. Storage root initiation and root-bulking rate, both complex traits, are highly relevant to breeders. However, measuring these critical traits and selecting varieties with improved root systems in the field is destructive, time-consuming, and expensive [14]. Instead, using a suitable controlled environment, root growth can be measured non-destructively over time. Genotypes with desired root architecture can be identified in the controlled environment and assessments of the selected genotypes can be made under field conditions to validate laboratory screens and assess field performance. Development of a simple, non-destructive root phenotyping method to capture root system architecture would facilitate identification of the mechanisms and factors controlling storage root related traits. This would increase understanding, allowing for alternative cropping systems as well as the optimization of varietal response to management practices [13].

Image-based plant phenotyping methods have gained more interest in recent years. The absence of high-throughput automated tools capable of providing accurate quantitative data on plant structure and function has been widely recognized as a key bottleneck to the production of better crops [17]. Image-based phenotyping can meet the requirements of breeders and other plant scientists and could allow a complete functional analysis of essential traits linking genotype to phenotype. However, accurate phenotyping requires carefully designed automated image acquisition hardware and software to be developed and embedded in reliable tools [18].

In this paper, we report on the development and testing of different root phenotyping platform designs to support the study of cassava RSA traits in real-time, and to identify the ideal system to monitor storage root (SR) development. We also discuss the ability to produce cassava storage roots in aeroponic systems, and the possibility of estimating root biomass using an image analysis platform. Finally, we have also validated the predictability of our developed aeroponic mist system using auxin experiments.

## Methods

### Model system testing and experimental conditions

To test the efficacy and efficiency of different systems on cassava storage root initiation and bulking we used the CIAT genebank accession MCOL1468, known for its good field performance and early root bulking. In order to select homogeneous plants for testing, we used in vitro plants from micro-cuttings developed at the CIAT plant tissue culture lab. The in vitro plants were transferred to the greenhouse for hardening using nutrient solution enriched with NO_3_^−^ (Additional file 1: Table S1), and after 20 days the plants were transferred to the respective model systems (semi-aeroponic, dripponic, and aeroponic) with a solution enriched with NH_4_NO_3_ (Additional file 1: Table S1). The N forms and the other nutrients (composition and concentration) were selected based on our preliminary cassava hydroponic root experiments [19] and other exploratory experiments before conducting the actual experiment here. The greenhouse conditions of these experiments were as follows: temperature ranges between 25 and 30 °C, average relative humidity 50% with natural sunlight and a photosynthetic flux of 1000–1200 μmol m^−2^ s^−1^. The nutrient solution was changed every 7 days, and the pH was maintained at 6.5 in all the systems tested. Since the current research aims to design a phenotyping system for early storage root initiation and bulking, we conducted all the experiments for up to 5 months. The experiments were conducted in a completely randomized design with five replications.

### Description of the different root phenotyping systems developed in this study

#### Semi-aeroponic system

**Fig. 1:**
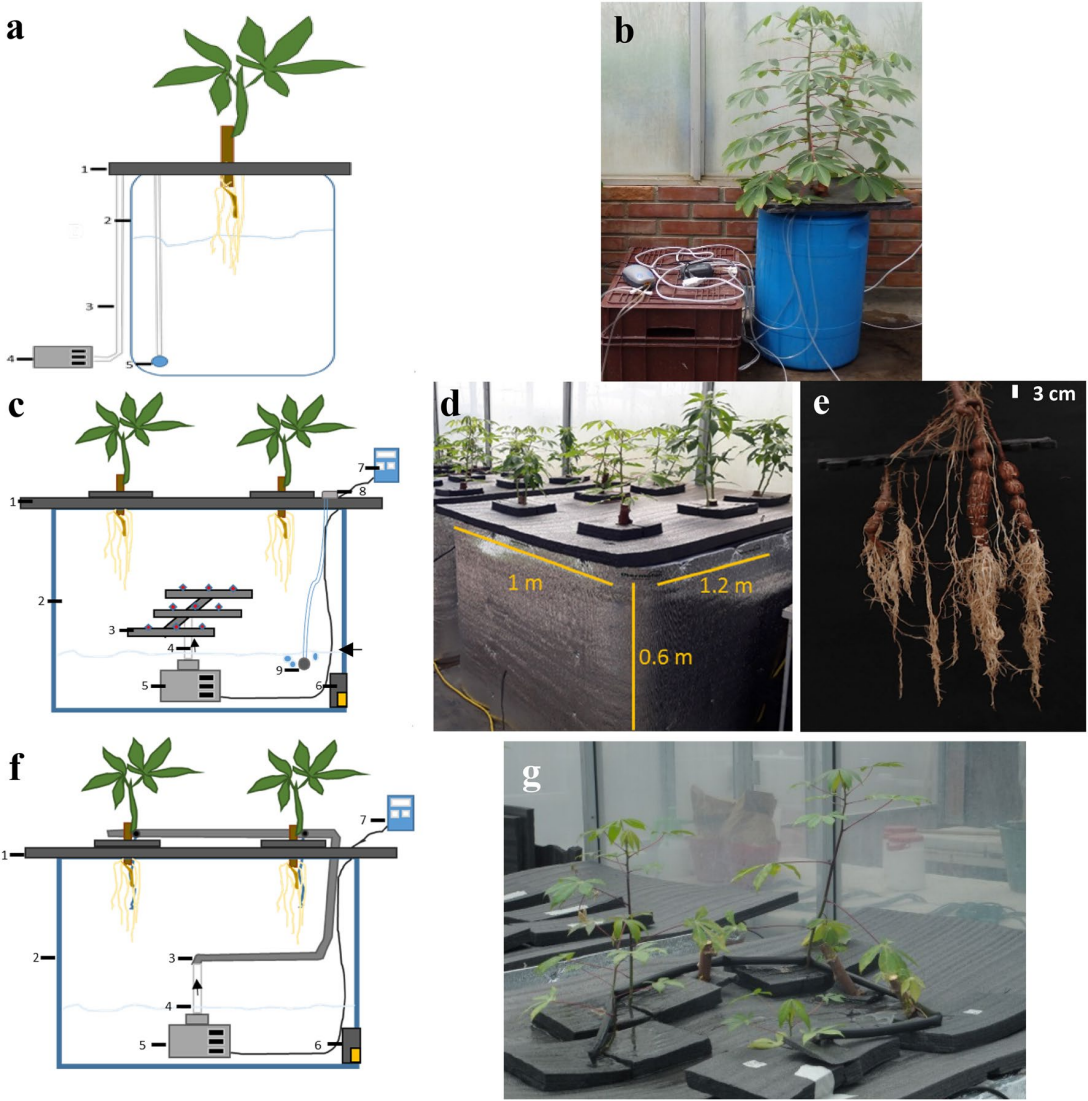
Schematic representation of different systems used for (SR) phenotyping. **a** Semi-aeroponic system 1. Foam Yumbolon 2. Container 3. Aquarium hose 4. Air pump 5. Aquarium air stone. **b** Full view of semi-aeroponic system. **c** Aeroponic mist system 1. Semi-rigid polystyrene foam 2. Container (Isotanque 100L) 3. Misting Spray 4. PVC tube 5. Submersible pump 6. Drain valve 7. Timer 8. Air pump 9. Aquarium air stone. **d** Full aeroponic system view. **e** Cassava root growth in the aeroponic system. **f** Dripponics System 1. Foam Yumbolon 2. Container (Isotanque 100L) 3. Flexible drip hose 4. PVC tube 5. Submersible pump 6. Drain valve 7. Timer. **g** Full view of dripponics system

The semi–aeroponic system consisted of a low-cost plastic bin, 60 × 35 × 66 cm (L × W × H; PLASTANK ltd, Colombia) (Fig. 1a, b) and covered with a black polystyrene foam (3 cm) to both hold in vitro plants and mask the root system from the light. Semi-aeroponics tanks were half-filled with 30 L of nutrient solution, in order to maintain semi-aerobic conditions. Throughout the study, the lower part of the root system was allowed to touch the nutrient solution and the upper part of the root system remained in the air to accelerate SR development (Fig. 1a). Aeration was supplied to the nutrient solution using the HP-200 magnetic vibration air pump (Sunsun Ltd., China). We sprayed NH_4_NO_3_ enriched solution manually using a hand mist sprayer (3 to 4 times per day) to the roots located in the upper part of the system to prevent dehydration (Fig. 1a).

#### Aeroponic mist system

Our custom-made aeroponic chamber system consists of a low-cost, recyclable container (Iso tank 1000 L) which measures 120 × 100 × 60 cm (L × W × H). We painted the plastic tank black to block the light, because SR development is sensitive to light. Then we wrapped the container with metallic polyester (Thermolon^®^) to minimize heat absorption (Fig. 1c, d). In order to ensure effective mist around the developing roots, this system used a 0.5 HP submersible pump, Aqua 60 W Evans (to pressurize the accumulator tank) which is connected through 3/4-inch PVC tubing to the sprinkler. The sprinkler mist system was adapted from the Clone King 64 mister. The P-200 magnetic vibration air pump (Sunsun Ltd., China) was used to aerate the solution (Fig. 1c). The nutrient flow through the aeroponics systems and timer cycle was programmed as follows: continuous flow for 24 h first 3 days after planting; then 4 to 8 days after planting the systems were programmed to sprinkle every 10 min for 30 s; and finally every 20 min for 20 s until harvest. The nutrient solution was changed every 7 days, and the pH was adjusted to 6.5. The black polystyrene semi-rigid foam called Yumbolon (Polylon SA, Colombia) (150 × 130 × 3 cm) was placed on top of the tank to hold the plants. Our current aeroponic system has six holes (20 × 20 cm), which can accommodate six plants per container (Fig. 1d, e). An additional piece of Yumbolon (25 × 25 cm) was used to more precisely support individual plants over the 20 × 20 cm holes.

#### Dripponics system

The dripponics system adopted the same structure as the aeroponic approach, but drip-delivered the nutrient solution instead of using a mist spray. The drip hose had a hole of approximately 0.8 mm to drip enough to wet the whole root system (Fig. 1f, g). The nutrient flow and timer cycle protocol were followed as in the aeroponics mist system described above.

### Validation of aeroponic mist system: auxin experiment

To validate the utility of our aeroponic mist system on SR phenotyping, we tested the effect of auxin on SR development in two contrasting cassava genotypes (GM3893-65 and MPER 183) which differ in root bulking rate. MPER 183 is an early bulking variety [20] while GM3893-65 is late root bulking under field conditions (Personal communication from Hernan Ceballos, Cassava Breeder, CIAT). The cassava stakes used in this experiment were obtained from the International Center for Tropical Agriculture (CIAT) germplasm collection maintained in the field. The experiment was conducted in a randomized block design with five replications with two auxin treatments using synthetic naphthalene acetic acid (NAA^+^ and NAA^−^). To select uniform planting material, we choose all the cuttings (stakes) from plants of the same age and of similar origin within a plant (primary stem), and with seven nodes. Initially, we treated the cutting with fungicide (Banrot 1 g L^−1^ for half an hour) to avoid fungal growth. Then the stakes were immersed in 1.5 g of NAA L^−1^ solution for 3 h from the distal end, leaving untreated the last three nodes at the proximal end. All the other experimental growth conditions were as described above in the aeroponic mist system.

### Field validation experiment

To confirm the aeroponic results conducted in the glasshouse, we conducted field experiments in 2018 at CIAT in Palmira, Valle del Cauca, at 3°29′ N and 76°21′ W, an altitude of 1020 m.a.s.l. General environmental conditions are: temperature varies from 19 to 30 °C; rainfall is bimodal, and soil texture is sandy clay loam. The same genotypes, hormone treatments, and experimental design were applied as in the aeroponics experiments. Weeds were controlled with hand weeding. Nutrients were maintained at an optimal level, and pests and diseases were controlled. Water was applied with an efficient drip irrigation system. Field experiments were harvested at the same time (5 months) as the aeroponic experiments.

### Agronomic trait measurement

The following agronomic data were recorded at time of harvest (5 months) from all the experiments conducted in this study: number of SR per plant, SR length, perimeter of SR, fresh and dry SR biomass, fresh and dry FR biomass, fresh and dry leaf biomass and SR volume (Archimedes method). Every day the roots were monitored visually and the number of days until storage root initiation from fibrous roots was recorded. Images of roots were also captured in the auxin experiments to correlate the data between ground-truths and images in order to develop the image analysis protocol. In the field experiment, we collected all the same traits except for days to storage root initiation, SR thickness and volume, since obtaining these data are a destructive and laborious process in field. However, random plants from the border rows were harvested to compare the root morphology between the aeroponic mist system and field.

### Histological experiment

To confirm the effect of the hormone on storage root initiation and bulking rate, we repeated the aeroponic experiments using two cassava genotypes (GM3893-65 and MPER 183), contrasting for late and early bulking, respectively, with four replicates and two treatments (NAA^+^ and NAA^−^) as in the aeroponic validation experiment described above. Once the storage roots began to thicken, two types of root samples (fibrous and storage) were taken from each treatment (NAA^+^ and NAA^−^): 12 fibrous and 6 storage root samples were randomly selected from each treatment. Next, the collected tissues were stained [21, 22] to determine the size and number of parenchyma and secondary xylem parenchyma cells. The histological pre-sample processing was performed according to [21, 23]; then the sections were stained with 1% safranin and 0.2% fast green and observed under light microscope (BX60; Olympus). Photos of each sample were taken using the imaging software Nikon-NIS Element F 4.0 and uploaded to the ImageJ software (Version, 1.8.0_112). We selected three different regions (square shape blocks) in the image from each of the two types of tissue and then counted and measured the cells.

### Root image capture and analysis

To capture the root images, we adopted a standard setup consisting of a semi-rigid polystyrene foam placed on a metallic support (1.6 m height from the floor) from which the plants hung freely. A black background (cloth) was placed behind this setup. A web camera (Logitech C922 Pro Stream) on a tripod was used to capture the images (Additional file 2: Fig. S1). While capturing the images, the plants were taken out of the aeroponic chamber and then quickly returned to the chamber to avoid dehydration. Height of the support and the distance of the camera from the plants remained constant while the images were captured.

Semi-automatic image analysis was carried out using the ImageJ software [24] (https://imagej.nih.gov/ij/download.html). First, the original image (Additional file 2: Fig. S2a) was loaded and transformed into HSV format, and manual segmentation was carried out to remove fibrous roots (FR) to highlight only the storage roots (Additional file 2: Fig. S2b). Then the images were transformed into 8-bit (greyscale), and the threshold was adjusted so that the storage roots were highlighted in black (Additional file 2: Fig. S2c). Afterwards, in the menu, we selected *particle analysis*; in the options, we chose *Outlines*; and in the particle size, we entered a number that allowed visualization of only the storage roots in the image (Additional file 2: Fig. S2d). Then this image was binarized using *fill hold* option, so that the storage roots were highlighted in black and the background was left white (Additional file 2: Fig. S2e). Finally, the processed image is saved before proceeding to the next steps. We installed SmartRoot [25] (https://smartroot.github.io/) in ImageJ. Then the previously processed image was loaded and with a semi-automatic and intuitive process, all the root images were selected and root measurements made (Additional file 2: Fig. S2f). Before analyzing the images, the program was calibrated using the reference (ruler) as follows: once the distance from the chamber to the roots was established, a reference (ruler) was placed in the root locus, and then an image was acquired, allowing pixel-based measurements to be converted to mm. Data obtained from the digital images were also correlated with manual measurements, in order to verify the accuracy and precision of the protocol.

### Statistical analysis

All statistical analyses were carried out with SAS 9.4 (SAS Institute, Cary, NC). The average values were compared using Fisher´s LSD test. The analyses mentioned above were performed using InfoStat [26] software. Statistical significance was set at the overall 0.05 level. Simple phenotypic correlations between the experiments were computed using Pearson correlations. The statistical analysis of the histological study was conducted using a 2 × 2 factorial design.

## Results

### System comparison

**Fig. 2:**
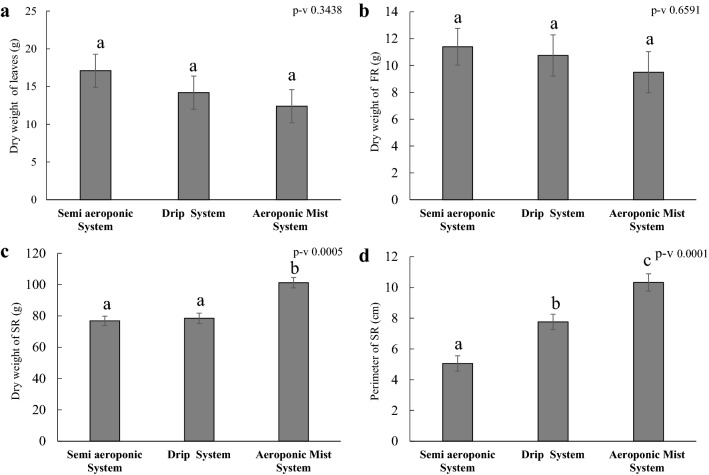
Growth performance of cassava in three different phenotyping systems. **a** Dry weight of the leaves, **b** dry weight of FR, **c** dry weight of the SR, **d** perimeter of the SR. Values are mean ± SE (n = 15)

Three different SR phenotyping systems were established to determine the most suitable system for cassava. All the tested systems developed storage roots but with different intensity. Since our focus was to select a sensitive SR phenotyping system that closely imitates real field conditions, we examined key parameters such as: storage root initiation, the rate of bulking, and storage root yield among the different systems (Fig. 2a–d). Our results revealed that the aeroponic mist system positively affected the development of the storage roots compared to other systems, showing significant positive differences compared to the other systems in variables such as storage root dry weight and root thickness (Fig. 2c, d). Storage root initiation was observed around 35 days after transfer to the aeroponic mist system (Additional file 1: Table S2). The different systems did not show significant differences in dry weight of leaves or fibrous roots (Fig. 2a, b).

The semi-aeroponic and dripponics systems have more simple set-ups and lower cost (Additional file 1: Table S3) compared to aeroponic mist, but the storage root morphology and development are much closer to field-grown plants in the aeroponic mist system (Additional file 2: Fig. S3). Storage roots were initiated 15–20 days earlier (Additional file 1: Table S2), and root bulking rate was faster (Fig. 2), in the aeroponic mist system as compared to the semi-aeroponic or dripponic systems. However, aeroponics is a little more expensive than the others since it needs sprinkler mist set up to spray nutrient solution.

### Predictability of aeroponic mist system—a case study from auxin experiments

**Fig. 3:**
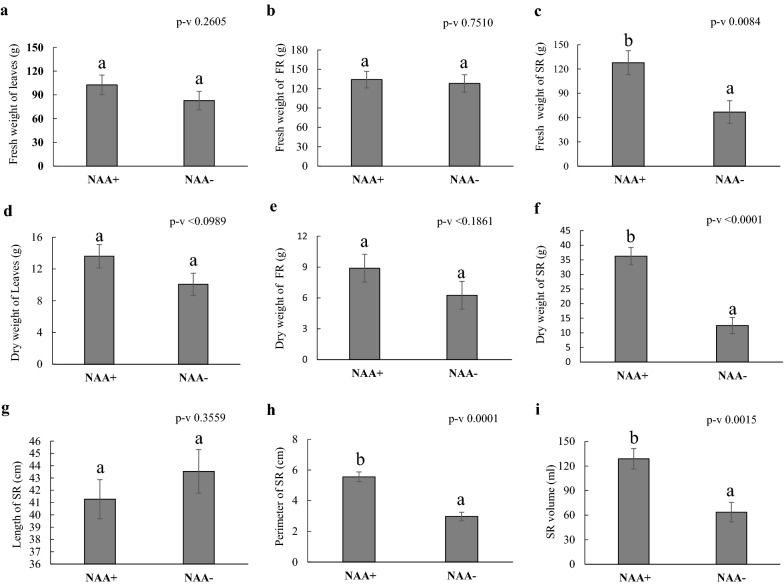
Effect of phytohormone (NAA) on cassava growth in aeroponic mist system. **a** Fresh weight of leaves, **b** fresh weight of FR, **c** Fresh weight of SR, **d** dry weight of the leaves, **e** dry weight of FR, **f** dry weight of SR, **g** length of the SR, **h** perimeter of the SR, **i** volume of the SR. Values are mean ± SE (n = 21)

To validate the predictability of the developed aeroponic mist system, we developed an experiment to observe the effect of auxin on shoot and root development (Fig. 3). When comparing root initiation with and without auxin treatment, we did not find any significant differences in days to FR initiation (Additional file 2: Fig. S4a). However, the stakes treated with NAA^+^ showed earlier storage root formation (Additional file 2: Fig. S4b) and more rapid bulking (Additional file 2: Fig. S5) compared to NAA^−^ treated stakes. We determined storage root initiation based on the color change (cream to dark brown) and thickness (Additional file 2: Fig. S6 a, b). Then the number of storage roots were counted. Fresh and dry storage root weight, and storage root thickness and volume (Fig. 3c, f, h, i) showed significant differences between the treatments. Interestingly, fresh and dry weight of the leaves, fibrous root weight and length did not show any significant differences (Fig. 3a, b, d, e, g). The results from this study showed that storage root growth was increased (fresh root weight by 91% and dry root weight by 190%) in the plants treated with auxin (NAA^+^) compared to non-treated (NAA^−^) (Fig. 3c, f).

**Fig. 4:**
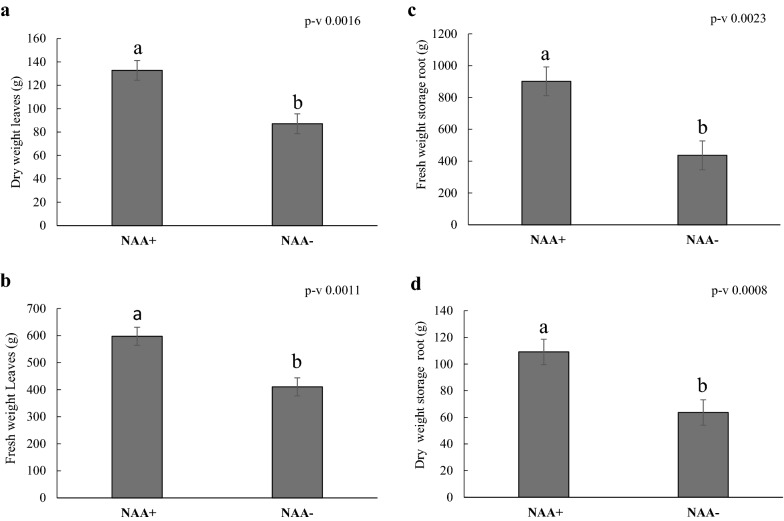
Effect of the phytohormone (NAA) on cassava growth in field conditions. **a** Fresh weight of the leaves, **b** dry weight of leaves **c** fresh weight of SR, **d** dry weight of the SR. Values are mean ± SE (n = 21)

The same auxin treatments were repeated in the field and the plants were harvested at the same age as in aeroponic mist, and results were compared (Fig. 4a–d). Our field results confirmed that stakes treated with NAA^+^ showed similar responses (Fig. 4c, d; Additional file 2: Fig. S7) in terms of fresh (106.5% increase compare to NAA^−^) and dry storage (71.5% increase compare to NAA^−^). In contrast, NAA^+^ treated field plants showed significant differences in fresh and dry leaf weight (Fig. 4a, b). Although we obtained similar responses on root traits to auxin in both environments, the storage root weight obtained from the field was higher than from the aeroponic mist system. The average dry weight of SR was around 109 g in the field compared to 36 g in aeroponic mist under auxin treatment.

### Histological study on auxin aeroponic mist experiment

**Fig. 5:**
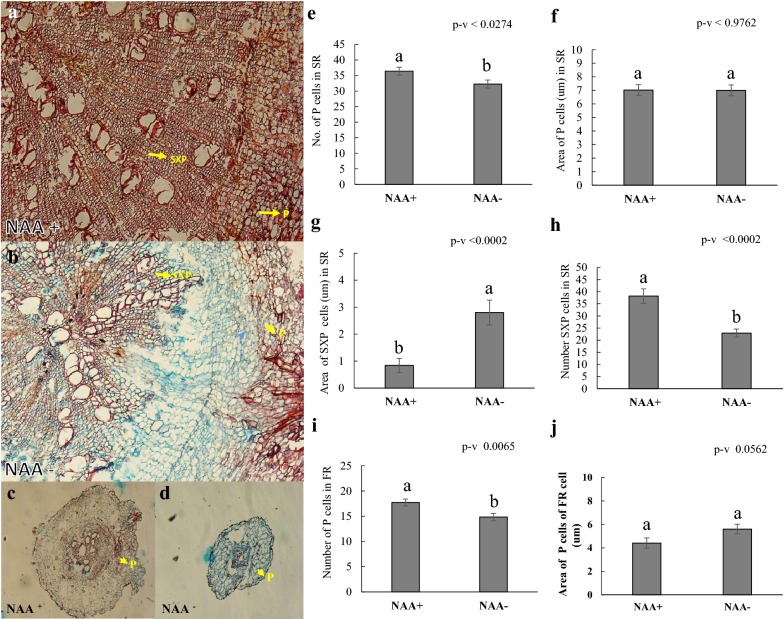
Histological studies of fibrous and storage root with and without Auxin (NAA^+^/NAA^−^) in the aeroponic mist system. **a** Cross section of SR obtained from the NAA^+^, **b** cross section of SR obtained from the NAA^−^, **c** cross section of FR obtained from the NAA^+^ **d** cross section of FR obtained from NAA^−^**, e** effect of auxin on the number of P cells in SR, **f** effect of auxin on the size of P cells in the SR, **g** effect of auxin on the size of secondary xylem parenchyma cells in SR, **h** effect of auxin on the number of secondary xylem parenchyma cells in the SR, **i** effect of auxin on the number of P cells in FR, **j** effect of auxin on the size of parenchyma cells in FR. Values are mean ± SE (n = 15)

To deepen our understanding of the effect of hormone regulation on storage roots (Fig. 5a–j), we made histological observations of the fibrous and storage roots. The histological results showed that the number of parenchyma cells in both storage and fibrous roots was significantly increased with the effect of auxin (Fig. 5a–e, i), while the size of the parenchyma cells did not show any significant differences (Fig. 5f, j). Also, we found that there was a significant increase in the number of secondary xylem parenchyma cells in plants treated with auxin (NAA^+^) (Fig. 5a, b, h), however, the size of these cells was smaller compared to the plants not treated with auxin (NAA^−^) (Fig. 5a, b, g).

### Development of high-throughput root phenotyping using image analysis

**Fig. 6:**
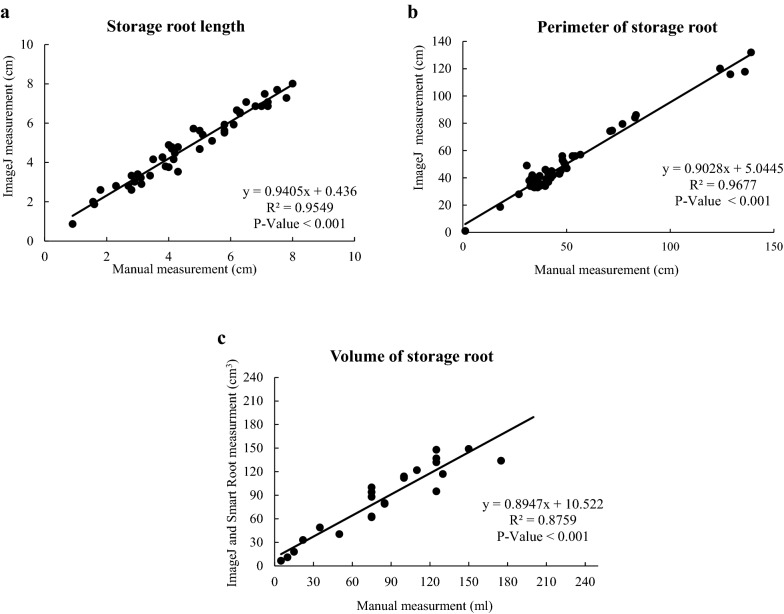
Correlation between manual and image based SR trait measurements. **a** SR length, **b** SR thickness, **c** SR volume

To maximize the efficiency of SR phenotyping, we developed a simple image analysis platform to estimate root length, perimeter and volume of the storage roots. To validate the method, we conducted a correlation analysis between image and manual measurements. Our Pearson correlation analysis revealed a highly significant positive correlation for root length, perimeter and volume of the SR, R^2^ = 0.95 (*p *< 0.001), R^2^ = 0.97 (*p *< 0.001) and R^2^ = 0.87 (*p *< 0.001), respectively (Fig. 6a–c). One of the challenges is to estimate the volume of complex 3D cassava root architecture using 2D images. This is mainly due to roots clumping or bunching together while taking images. However, this issue was resolved using simple polyether foam separators to distribute the roots while taking the pictures (Additional file 2: Fig. S1), preventing roots from overlapping. This simple root distribution helps us to analyze 2D images through the ImageJ and SmartRoot software to estimate the volume, length, and perimeter throughout the crop cycle with a high degree of precision.

## Discussion

### Aeroponic mist is an ideal system to study cassava storage roots

There is broad interest in developing a cassava root phenotyping platform to study RSA traits under controlled environmental conditions, which are predictive of field performance. However, controlled environment-based studies in cassava are few [27], and no specific storage root phenotyping method exists. ‘Shovelomics’ (uncovering roots or uprooting plants in the field) in cassava is laborious and requires destructive sampling; real-time monitoring is almost impossible. The heterogeneity in the soil physical and chemical composition that can affect the RSA of field-grown cassava at different locations is another factor confounding the effect, due to G × E [28]. The methods exploited to visualize RSA vary from one crop to another, based mainly on the crop duration, root morphology, and root function. Most advances in root phenotyping methods were achieved in cereals such as rice. The root system architecture of these crops is not very complex, and they are easy to grow hydroponically [9, 20]. However, root phenotyping of root crops like cassava is difficult due to complexities such as multiple functionality—e.g. storage, anchoring and seeking/uptake of water and nutrients.

In the present study, we attempted to design a low-cost and sensitive platform able to support studies of cassava root system architecture. Since storage roots did not bulk in the fully submerged conditions (hydroponics) in previous studies, we attempted different methods to obtain storage roots. Initially, we constructed semi-aeroponic systems to establish the SR development protocol. Based on our previous [19] and preliminary research, we found that four main factors (nutrients, air, light, and mist pressure on storage roots) contribute to storage root formation under controlled conditions.

Nutrients: Through our preliminary studies [19], we have found that nitrogen forms were influencing storage root initiation and bulking. Since cassava is an aerobic crop, we used a NO_3_^−^ enriched nutrient solution to induce roots. When we continued to use the sole NO_3_^−^ N enriched solution in the experiment, we did not obtain any storage root bulking; the only effect noted was that FR became elongated. Our nitrogen nutrient studies confirmed that NO_3_^−^ enhanced early fibrous root vigor (first 30 days); however, the roots were sensitive to sole NH_4_^+^ enriched nutrient solution. When we switched to NH_4_NO_3_ (50–50%) enriched nutrient solution in the system, we started to observe storage root initiation and bulking. Different N sources (NH_4_^+^, NO_3_^−^) and their combination (NH_4_NO_3_) were tested to improve plant growth reported in potato [29, 30]. Our study also confirmed that storage root initiation was induced when we switched to an NH_4_NO_3_ (50–50%) enriched solution. Several authors have reported the influence of nitrogen sources on crop growth and root vigor [31, 32]. Ammonium ions (NH4^+^) supplied as the sole nitrogen source inhibited crop growth compared to NH_4_NO_3_ and sole NO3^−^ [33, 34]. Crop growth seems to improve when a combination of NH_4_^+^ and NO_3_^−^ is taken up by the plant.

Air: SR initiation and bulking need aeration; submerged (hydroponic) conditions did not induce SR bulking.

Light: The storage root bulking process needs darkness. Gentle mist pressure: Although the semi-aeroponic system was successful in developing storage roots, we found issues of late root initiation and bulking which do not reflect the field reality. To improve the system further, we tested the dripponics system. The drip system had a minor effect on the development of the storage roots compared to the aeroponic spray system (Fig. 2). The drip system also did not produce any pressure on the growing roots, mainly because the water flow had little contact with a large part of the surface area of the root and therefore reduced the uptake of nutrients and oxygen [35]. However, in the case of the aeroponic mist system, regular mist cycles keep the roots moist and avoid drying out, as well as providing the consistent nutrients with gentle mist pressure.

Our aeroponics system uses a mist of nutrients over the roots inside a dark growth chamber. The consistent mist spray interval and gentle mist pressure on roots helps to initiate the storage roots faster, as long as the root chamber is kept at ideal temperatures. We recorded and monitored the chamber temperature periodically, and we observed that the noon-time chamber temperature reached up to 31 °C, depending on the greenhouse temperature and climate. However, the storage root development of cassava was normal even at somewhat elevated temperature; we did not notice any root death. Usually in aeroponics systems, when moisture is deficient in the root zone, plants can begin to senesce and transpire less water. Therefore, we ensured that the misting schedule would prevent drying of the roots; the chamber was statically controlled for mist for 30 s every 20 min.

Some advantages of our aeroponic mist system are that it is custom-made, is low-cost, uses nutrient solution efficiently, and provides roots with maximum oxygen. It is closer to the soil environment than hydroponics and the crops grow more rapidly [36]. In addition, the aeroponic mist setup described in this paper enables easy and local root sampling, without destroying the rest of the plant. Time series experiments are thus feasible, e.g., for cytology or gene-expression analyses which lead to novel gene identification regulating storage root yield in cassava.

### NAA seed treatment has the potential to accelerate root bulking

To prove the utility of the developed aeroponic mist system, we carried out a study to explore some of the key questions still unanswered: what makes cassava roots initiate the starch storage process, and what are the key factors that enhance this critical process?. Based on earlier results on other root crops [37, 38], we hypothesized that auxin has potential to accelerate starch storage in cassava in an aeroponic system. Our aeroponic mist system demonstrated the influence of NAA on root development (Fig. 3). Auxin may have several functions in the initiation, bulking and growth of storage roots [39]. In the present study, NAA-treated plants were significantly different in terms of storage root differentiation (Additional file 2: Fig. S4), bulking (Additional file 2: Fig. S5) and storage root yield than non-treated (Fig. 3c, f).

To test the predictability of the aeroponics system, we also confirmed results through a field experiment. Our field experiment results revealed that storage root yields obtained from the field grown plants were higher compared to the aeroponic-grown plants (Figs. 3f, 4d). However, the genotype responses to auxin effect on SR were the same as those obtained from aeroponics, which indicates the predictability of the aeroponic mist system. It is evident that the aeroponic system lacks interaction with the soil and other aspects such as mechanical, electrochemical and biological factors that affect the developing roots [40, 41], so it is logical to suggest that the lack of these interactions could explain the higher yields in the field. However, we observed that the time and mode of differentiation from fibrous roots to storage roots was almost the same in both aeroponics and field situations (Additional file 2: Fig. S6, S7).

Our histological results showed that the number of parenchymatic and secondary xylem parenchyma cells were significantly increased in auxin-treated (NAA^+^) plants (Fig. 5a–j). This increase resulted in earlier thickening and bulking than in non-treated plants (NAA^−^). The bulking of a fibrous root destined to become a storage root happens through secondary growth development, causing the proliferation of secondary xylem parenchyma in which starch is stored. Taken together, these results demonstrated that auxin plays a role in the formation of storage roots by activating the proliferation of parenchymatic and secondary xylem parenchyma cells to induce the initial thickening growth of storage roots. This finding agrees with results reported in earlier studies of sweet potato [42]. Noh et al. [42] reported that a MADS-box protein copy DNA, SRD1 boosts the proliferation of the metaxylem and cambium cells through the auxin-dependent initial bulking and growth of storage roots. However, no literature was available on the genetic control of adventitious and lateral roots in cassava [14], due to the lack of a suitable model system to study those mechanisms.

### Towards high-throughput root image analysis platform

To provide a complete package to the cassava root research community, we also developed a simple image analysis protocol to estimate storage root traits in real-time. We used the ImageJ program because it is open access, reliable and easy to use [43]. Similarly, SmartRoot is open access, and is a very intuitive, semi-automatic tool with better management of output files compared to other programs [44]. The primary objective of this process is to estimate length, diameter and volume of storage roots without sacrificing plants, thus allowing us to study the root dynamics over time. The semi-automatic image analysis developed from this research takes approximately 50 s per photo using macros. We utilized the color to classify storage (dark brown) and fibrous (light) roots [45]. However, it is important to mention that some SR can have a color very similar to that of FR; in this case, the segmentation was done manually based on root thickness, which took more time. In the future, it’s viable to develop machine learning models to differentiate storage roots based on color change and thickness. We used natural light as a source of illumination when acquiring images, and it is essential to bear in mind that this light is subject to natural variations that can change the characteristics of the image, which affects its subsequent analysis [46]. We solved this issue by taking the images during a fixed hour of the day (in the morning), and we also always took a photograph of a standard reference to adjust to a standard illumination level. Cassava roots are complex structures comprising fibrous and storage roots. The number and size of the storage roots are potential phenotypic traits reflecting crop yield and quality. Counting and measuring the size of cassava storage roots is usually done manually or semi-automatically, by first segmenting cassava root images. However, occlusion of both storage and fibrous roots makes the process both time-consuming and error-prone. There is a need for more automated storage root image analysis approaches to support the current phenotyping hardware, making the analysis more high-throughput. We will investigate machine learning approaches to remove some of the manual steps from the current procedure. Our ultimate aim is to develop a fully-automated, image-based phenotyping system to automatically count storage roots in cassava, including early bulking storage roots (usually from 0 to 2.5 months). The developed root phenotyping systems and image analysis protocols from this study can also be easily transferable to other root and tuber crops.

## Conclusion

A key goal in developing the aeroponics mist system were: (1) to have a product; and (2) to have the capacity for reliable high throughput, non-destructive phenotyping of root systems. We presented here a simple, low-cost aeroponic mist system that allows time-series analysis of storage root initiation up to critical stages of early SRD. Root sampling using our aeroponic mist system is easy and practical for molecular and microscopic analyses. As such, the cassava aeroponics setup opens new avenues for in-depth storage root research. This study also provides novel insights into auxin interactions. These experimental findings have practical applications in cassava production, to accelerate the storage root bulking using simple hormone treatments. The results of the current study point to follow-up work that investigates the major gene(s) regulating the auxin pathway in cassava. Evidence suggests that there must be a differential gene expression in the two root types. Using the developed aeroponic mist system should help identification of these genes, and ultimately improve yield. Cassava is “sink-limited” and the current research will allow us to study the genes controlling the cassava sink-related traits. This research has profound implications for cassava genetic improvement and sustainable intensification. The output of the current research may lead to finding a novel gene/factor with significant control over rate of starch accumulation in cassava storage roots.

## Supplementary information

**Additional file 1: Figure S1.** Root image acquisition platform. 1. Semi-rigid polystyrene foam 2. Metallic support 3. Root separator 4. Root system 5. Camera 6. Cloth black background 7. Tripod. **Figure S2.** Work flow of Root image analysis. (a) Original image (b) Segmentation of the FR (c) Enhancement and binarization of SR (d) Selection of SR using particle size (e) Image with enhanced SR (f) Image processed with SmartRoot. **Figure S3.** Comparison of SR development grown under aeroponic and field conditions at the same age. **Figure S4.** Effect of auxin on FR initiation and SR differentiation. (a) Effect of auxin on FR initiation, (b) Effect of auxin on the SR differentiation (number of fibrous roots changed the color to begin bulking). Values are mean ± SE (n = 15). **Figure S5.** Time series analysis of SR bulking of MPER 183 and GM3893-65 under (NAA^+^) and (NAA^−^) treatments.). Values are means from six plants from each genotype. **Figure S6.** Comparison of cassava root initiation and development in aeroponic and field grown plants at 50 days after planting. (a) Cassava roots grown in aeroponics system, (b) Cassava roots grown in the field. The yellow circles indicates the FR which are transformed to SR with ramification. Arrows shows the differentiation of FR changing color and thickness before transforming as SR. The green circles shows FR without root ramifications. **Figure S7.** Effect of auxin (NAA) on root initiation and bulking in aeroponics mist system and field conditions.

**Additional file 2: Table S1.** The composition of nutrient solution used in this study. **Table S2.** Variation of storage root initiation under different phenotyping systems. Values are mean from six plants. **Table S3.** Cost comparison of different root phenotyping systems developed in this study.

## Data Availability

The data used in this study is available from the corresponding author on reasonable request.

## Abbreviations

SR: storage root
SRD: storage root development
FR: fibrous roots
RSA: root system architecture
NAA: naphthalene acetic acid
