# Supplementary material for: A low-cost aeroponic phenotyping system for storage root development: unravelling the below-ground secrets of cassava (Manihot esculenta)
Source: Plant Methods. 2019 Nov 9;15:131. doi: 10.1186/s13007-019-0517-6 (PMC6842211; doi:10.1186/s13007-019-0517-6)
Supplement: Supplementary file 1 — Additional file 1: Figure S1. Root image acquisition platform. 1. Semi-rigid polystyrene foam 2. Metallic support 3. Root separator 4. Root system 5. Camera 6. Cloth black background 7. Tripod. Figure S2. Work flow of Root image analysis. (a) Original image (b) Segmentation of the FR (c) Enhancement and binarization of SR (d) Selection of SR using particle size (e) Image with enhanced SR (f) Image processed with SmartRoot. Figure S3. Comparison of SR development grown under aeroponic and field conditions at the same age. Figure S4. Effect of auxin on FR initiation and SR differentiation. (a) Effect of auxin on FR initiation, (b) Effect of auxin on the SR differentiation (number of fibrous roots changed the color to begin bulking). Values are mean ± SE (n = 15). Figure S5. Time series analysis of SR bulking of MPER 183 and GM3893-65 under (NAA+) and (NAA−) treatments.). Values are means from six plants from each genotype. Figure S6. Comparison of cassava root initiation and development in aeroponic and field grown plants at 50 days after planting. (a) Cassava roots grown in aeroponics system, (b) Cassava roots grown in the field. The yellow circles indicates the FR which are transformed to SR with ramification. Arrows shows the differentiation of FR changing color and thickness before transforming as SR. The green circles shows FR without root ramifications. Figure S7. Effect of auxin (NAA) on root initiation and bulking in aeroponics mist system and field conditions. [file 13007_2019_517_MOESM1_ESM.docx]

| **Nutrient solution enriched with NO_3_^-^** | **Formula Weight** | **Concentration in solution** |
| --- | --- | --- |
| Potassium Nitrate: KNO_3_ | 101.1 | 0.3 mM |
| Sodium Phosphate; Na_2_HPO_4_ | 142 | 0.18 mM |
| Calcium Chloride Dehydrate; CaCl_2_ 2H_2_O | 147 | 0.36 mM |
| Magnesium Sulfate Heptahydrate; MgSO_4_ 7H_2_O, | 246.5 | 0.46 mM |
| EDTA | 367.1 | 45 uM |
| Boric acid; H_3_BO_3_ | 61.83 | 18 uM |
| Manganese(II) Sulphate Monohydrate; MnSO_4_ H_2_O | 169 | 4.6 uM |
| Zinc Sulfate Heptahydrate; ZnSO_4_ 7H_2_O | 287.5 | 1.5 uM |
| Cupric Sulfate; CuSO_4_ | 249.7 | 1.5 uM |
| Sodium molybdate; Na_2_MoO_4_ | 242 | 1 uM |
|  |  |  |
|  |  |  |
| **Nutrient solution enriched with NH_4_^+^** | **Formula Weight** | **Concentration in solution** |
| Ammonium Sulfate; (NH_4_)2SO_4_ | 132.1 | 0.3 mM |
| Potassium Sulfate; K_2_SO_4_ | 174.3 | 0.3 mM |
| Sodium Phosphate; Na_2_HPO_4_ | 142 | 0.18 mM |
| Calcium Chloride Dehydrate; CaCl_2_ 2H_2_O | 147 | 0.36 mM |
| Magnesium Sulfate Heptahydrate; MgSO_4_ 7H_2_O, | 246.5 | 0.46 mM |
| EDTA | 367.1 | 45 uM |
| Boric acid; H_3_BO_3_ | 61.83 | 18 uM |
| Manganese(II) Sulphate Monohydrate; MnSO_4_ H_2_O | 169 | 4.6 uM |
| Zinc Sulfate Heptahydrate; ZnSO_4_ 7H_2_O | 287.5 | 1.5 uM |
| Cupric Sulfate; CuSO_4_ | 249.7 | 1.5 uM |
| Sodium molybdate; Na_2_MoO_4_ | 242 | 1 uM |

**Table S1**.  Composition of nutrient solution used in this study

**Table S2.** Variation of storage root initiation under different phenotyping systems. DAST values are mean from six plants.

| **System** | **DAST*** | **Method used** |
| --- | --- | --- |
| Semi-Aeroponic | 55 | Visual color change and thickness |
| Dripponic | 50 | Visual color change and thickness |
| Aeroponic mist | 35 | Visual color change and thickness |

* Days after the stakes transferred (DAST)

**Table S3.**  Cost comparison of different root phenotyping systems developed in this study.

| **System** | **Cost per unit (USD)** | **No. of plant accommodated per unit** |
| --- | --- | --- |
| Dripponic | 150 | 6 - 8 |
| Aeroponic mist | 180 | 6 - 8 |
| Semi-aeroponic | 25 | 1 |
| Clone king Aeroponic system | 240 | 1 |
