# Supplementary material for: A low-cost aeroponic phenotyping system for storage root development: unravelling the below-ground secrets of cassava (Manihot esculenta)
Source: Plant Methods. 2019 Nov 9;15:131. doi: 10.1186/s13007-019-0517-6 (PMC6842211; doi:10.1186/s13007-019-0517-6)
Supplement: Supplementary file 2 — Additional file 2: Table S1. The composition of nutrient solution used in this study. Table S2. Variation of storage root initiation under different phenotyping systems. Values are mean from six plants. Table S3. Cost comparison of different root phenotyping systems developed in this study. [file 13007_2019_517_MOESM2_ESM.pptx]

## Slide 1
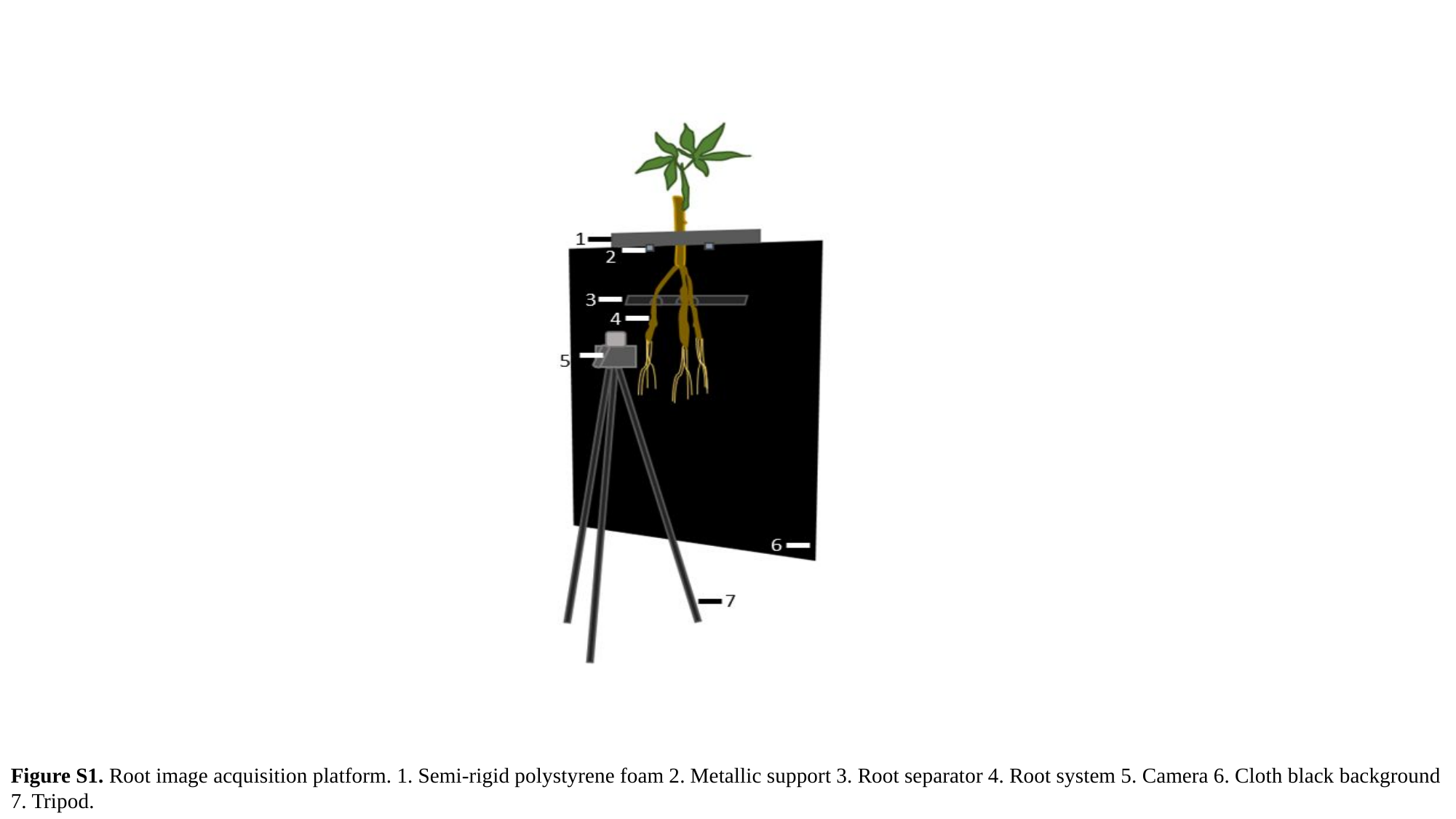

Figure S1. Root image acquisition platform. 1. Semi-rigid polystyrene foam 2. Metallic support 3. Root separator 4. Root system 5. Camera 6. Cloth black background 7. Tripod.

## Slide 2
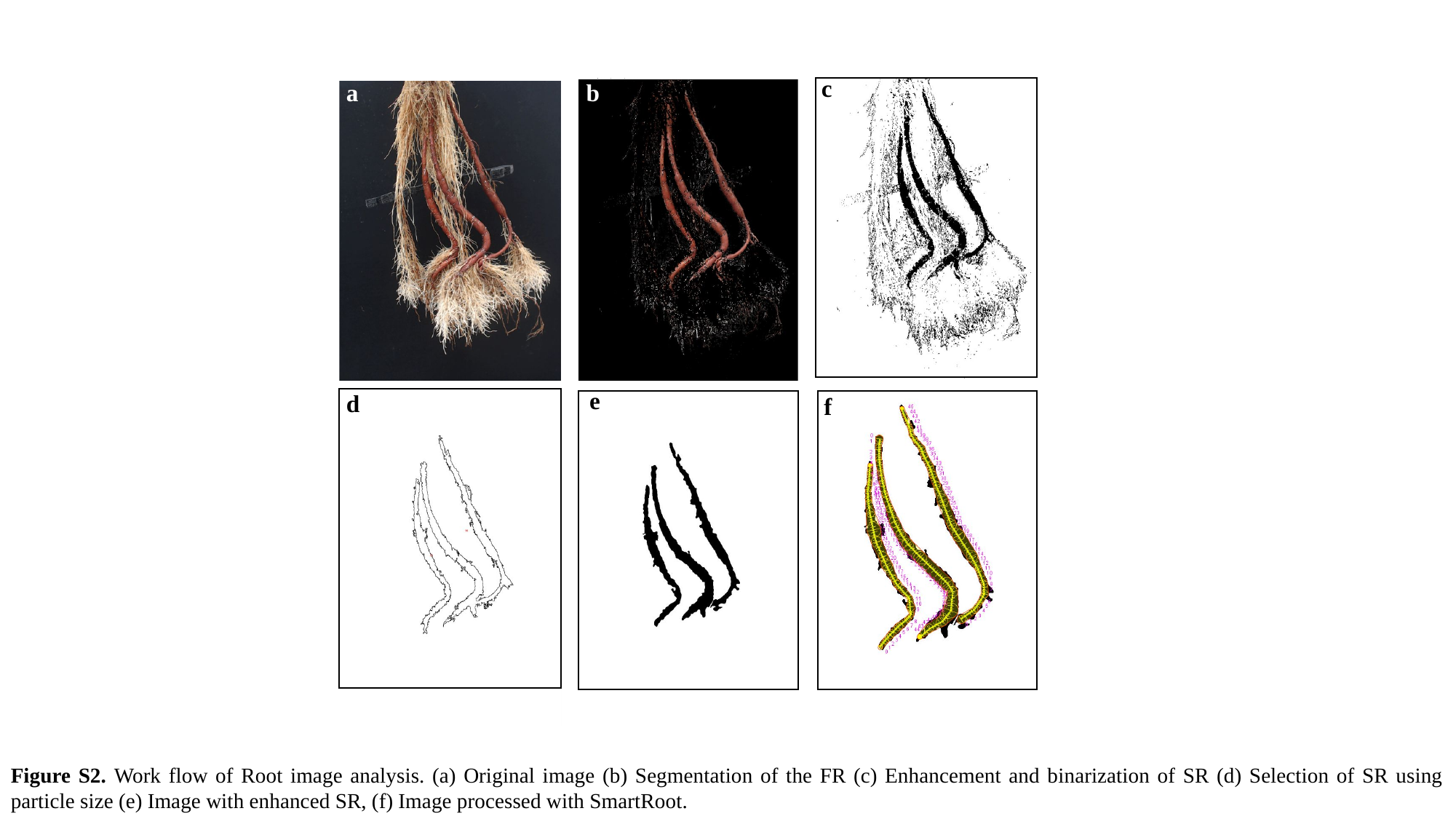

c
a
b
e
d
f
Figure S2. Work flow of Root image analysis. (a) Original image (b) Segmentation of the FR (c) Enhancement and binarization of SR (d) Selection of SR using particle size (e) Image with enhanced SR, (f) Image processed with SmartRoot.

## Slide 3
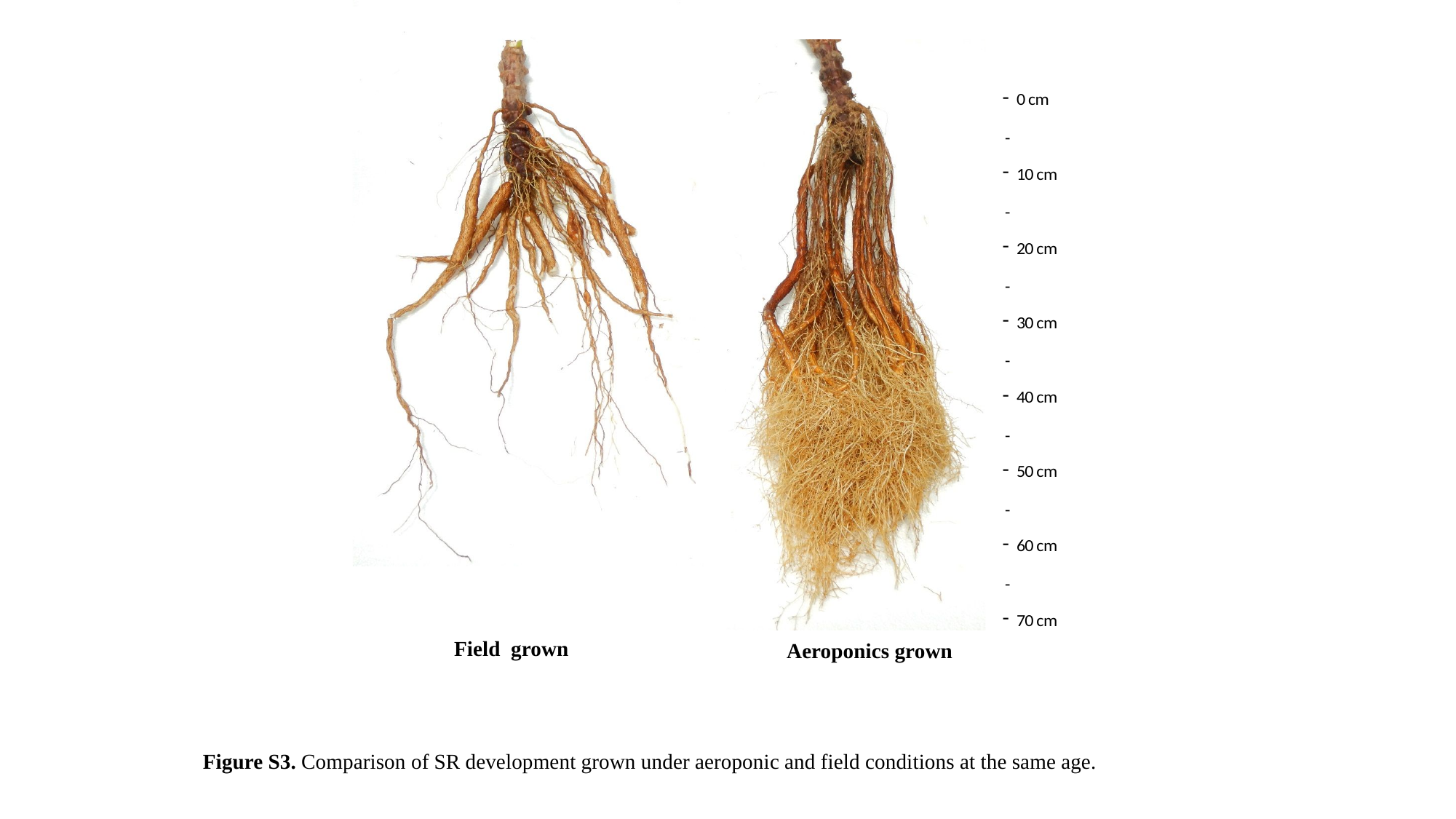

Field  grown
Aeroponics grown
Figure S3. Comparison of SR development grown under aeroponic and field conditions at the same age.

## Slide 4
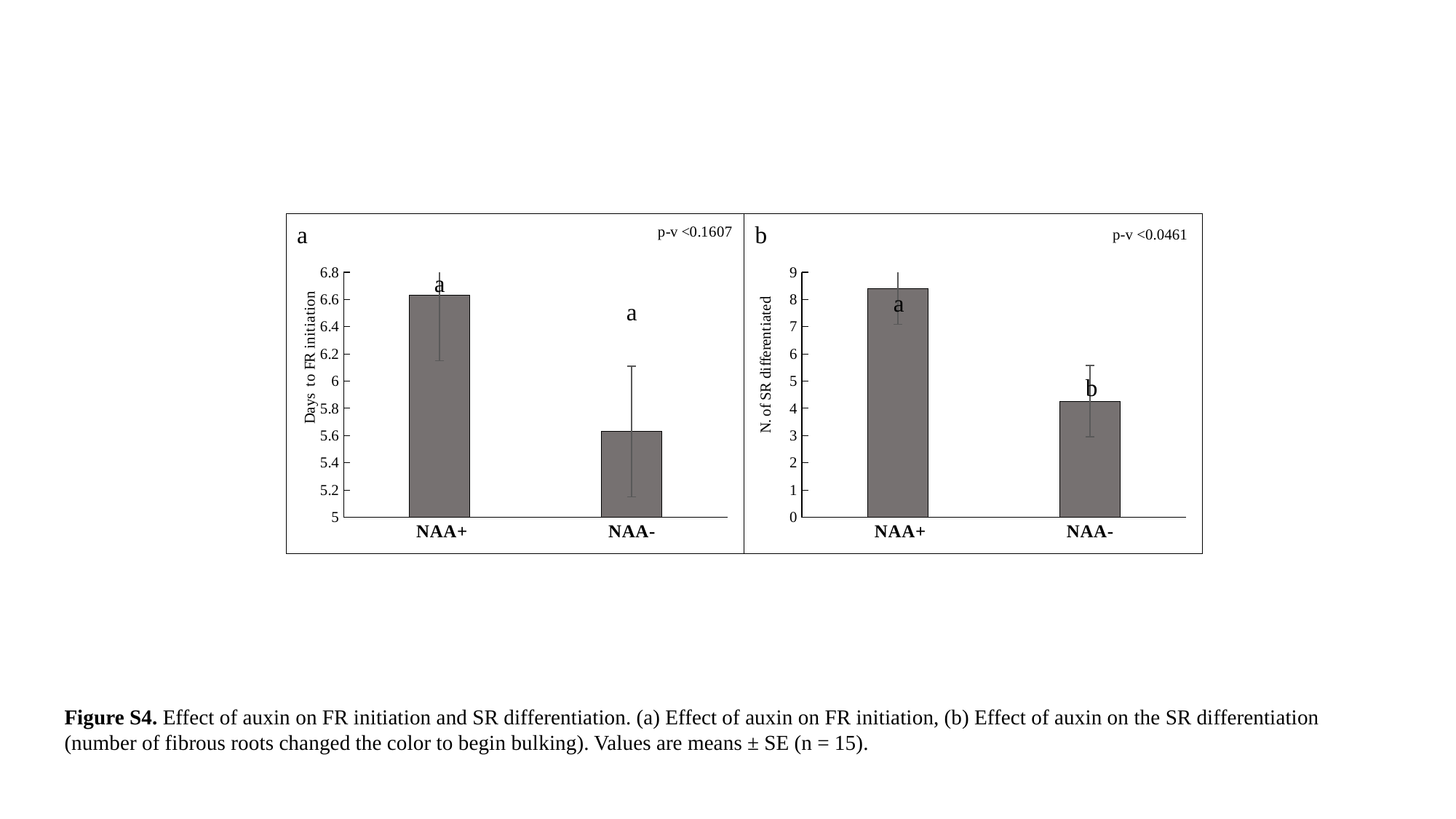

### Chart
| Category | |
|---|---|
| NAA+ | 6.63 |
| NAA- | 5.63 |a
### Chart
| Category | |
|---|---|
| NAA+ | 8.4 |
| NAA- | 4.26 |p-v <0.0461
b
Figure S4. Effect of auxin on FR initiation and SR differentiation. (a) Effect of auxin on FR initiation, (b) Effect of auxin on the SR differentiation (number of fibrous roots changed the color to begin bulking). Values are means ± SE (n = 15).

## Slide 5
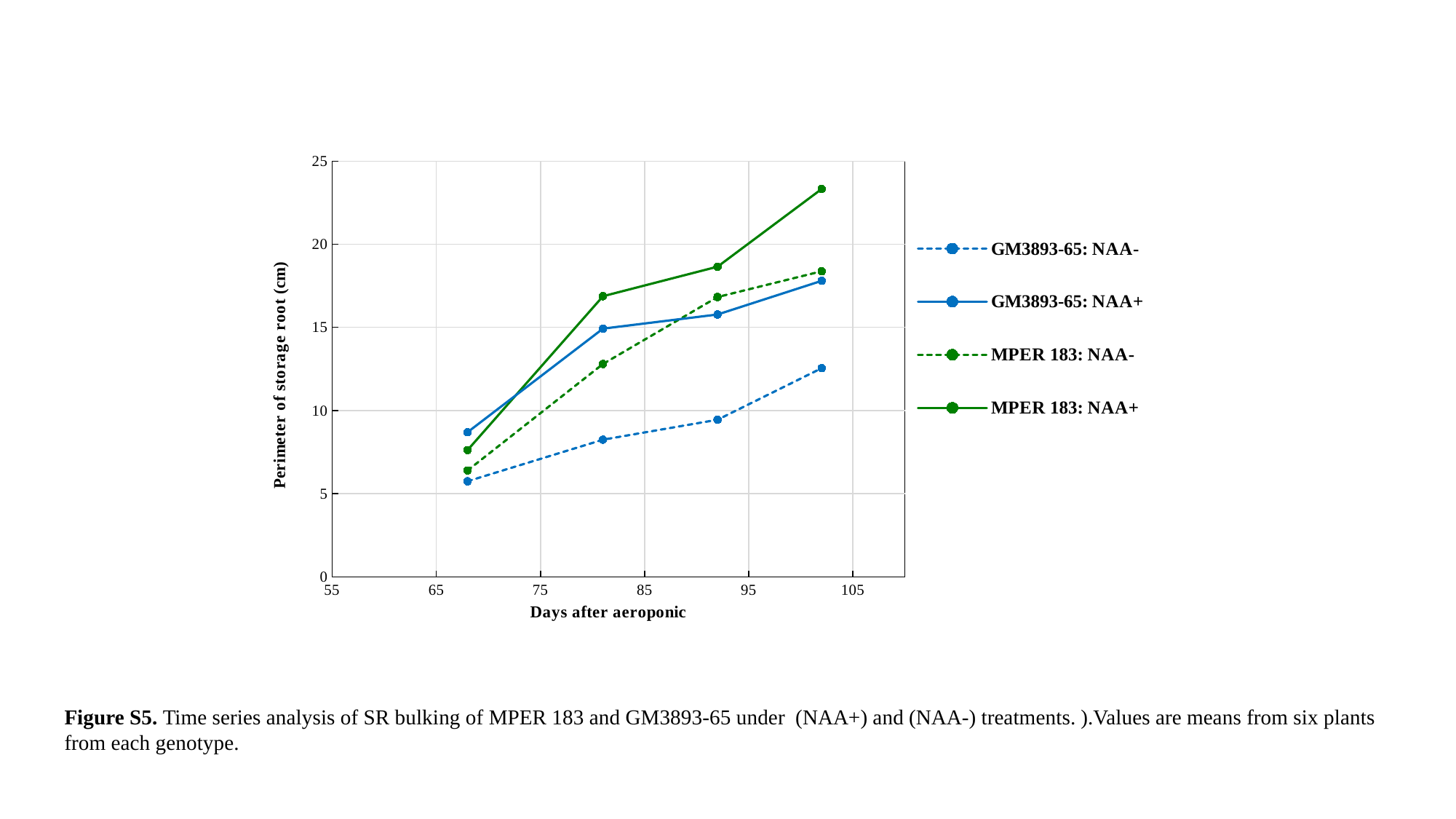

### Chart
| Category | GM3893-65: NAA- | GM3893-65: NAA+ | MPER 183: NAA- | MPER 183: NAA+ |
|---|---|---|---|---|Figure S5. Time series analysis of SR bulking of MPER 183 and GM3893-65 under (NAA+) and (NAA-) treatments. ).Values are means from six plants from each genotype.

## Slide 6
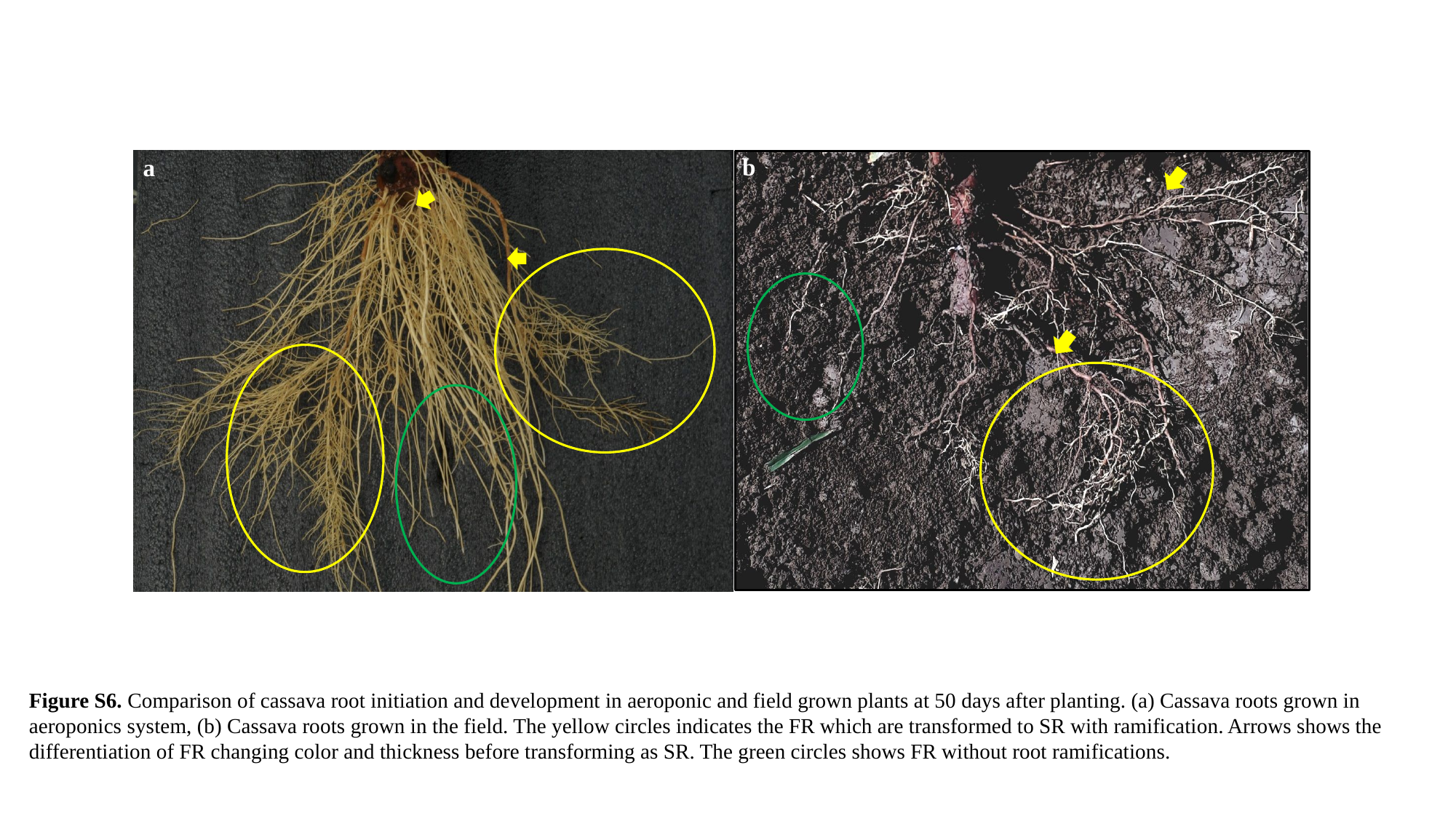

b
a
Figure S6. Comparison of cassava root initiation and development in aeroponic and field grown plants at 50 days after planting. (a) Cassava roots grown in aeroponics system, (b) Cassava roots grown in the field. The yellow circles indicates the FR which are transformed to SR with ramification. Arrows shows the differentiation of FR changing color and thickness before transforming as SR. The green circles shows FR without root ramifications.

## Slide 7
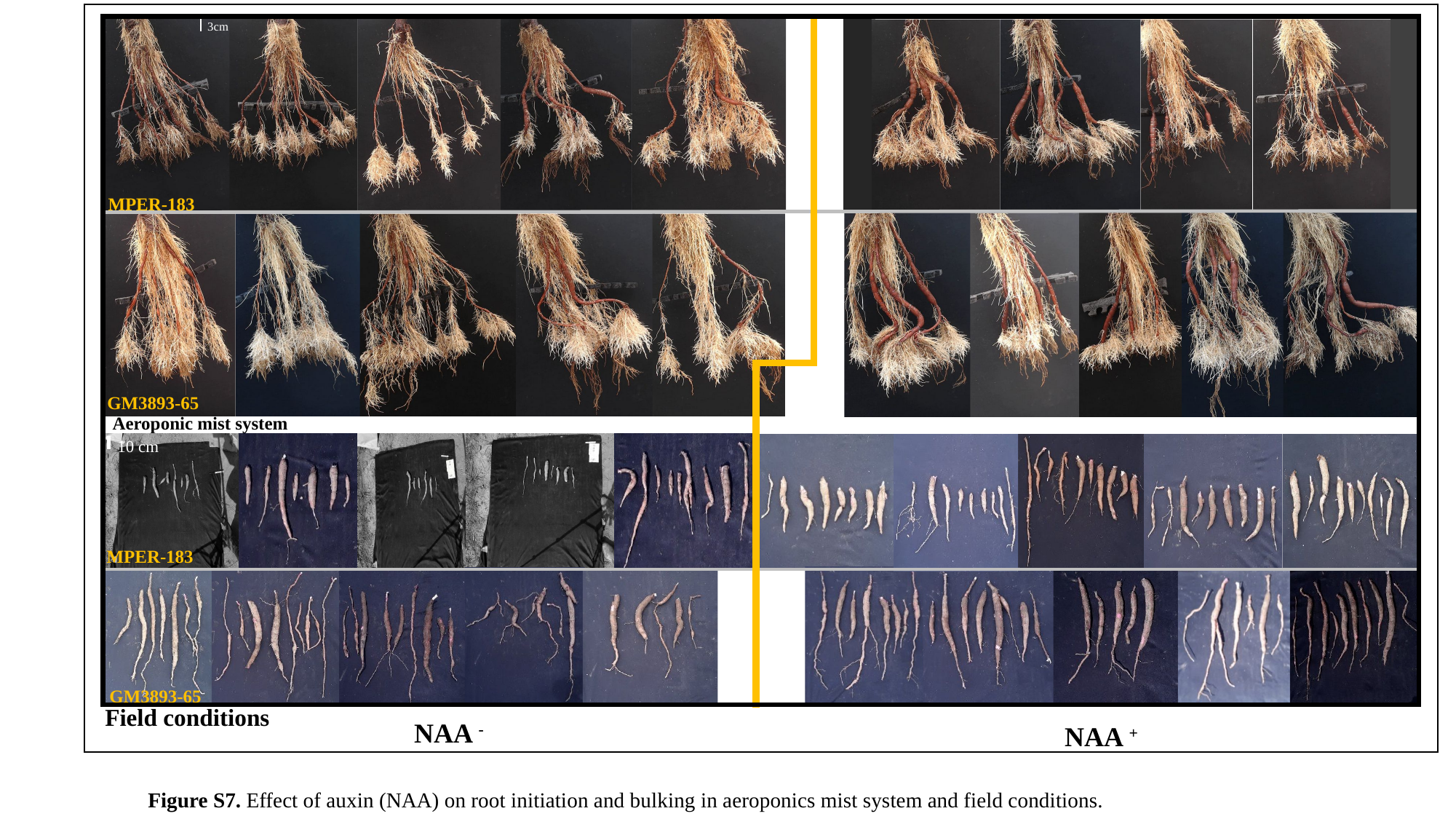

3cm
MPER-183
GM3893-65
Aeroponic mist system
10 cm
MPER-183
GM3893-65
Field conditions
NAA -
NAA +
Figure S7. Effect of auxin (NAA) on root initiation and bulking in aeroponics mist system and field conditions.
